# Pigeons in a flock go cheap: a re-evaluation of the energetics of flying in cluster flocks

**DOI:** 10.1098/rsbl.2025.0031

**Published:** 2025-07-09

**Authors:** Charles M. Bishop, Lewis G. Halsey, Graham N. Askew

**Affiliations:** ^1^ School of Environmental and Natural Sciences, Bangor University, Bangor, Gwynedd, UK; ^2^ School of Life and Health Sciences, University of Roehampton, London, UK; ^3^ School of Biomedical Sciences, University of Leeds, Leeds, West Yorkshire, UK

**Keywords:** flock flight, birds, energy expenditure, acceleration, heart rate

## Abstract

The energy expended by animals during locomotion is often of considerable ecological importance. However, inaccurate conclusions about energy expenditure may arise if the limitations of the proxy variables being used to infer locomotion costs are not considered. The study of ‘cluster’ flocking behaviour in pigeons, using wingbeat frequency as a proxy for mechanical energy output, provides a useful illustration of these pitfalls. In contrast to claims in the literature, we suggest that published body kinematic values measured with accelerometers, along with our own heart rate data, show little evidence in support of the hypothesis that there is an increase in the mechanical energy costs for most pigeons to fly in a typical ‘cluster’ flock. Indeed, our re-analyses of acceleration-based measures of body power suggest there may be a positive energetic advantage to flying at low flock densities. We suggest that, when assessing energy expenditure using accelerometry-derived variables, the energy proxy units should be consistent with those of power, whether mass specific or absolute, and should control for differences in body mass and speed where appropriate. Accumulated total journey costs should also be assessed alongside instantaneous costs, with the former likely to be at least as ecologically significant.

## 1. Introduction

Energy consumed during locomotion can represent a substantial portion of an animal’s daily energy budget [1–6]. For this reason and others, there is much interest in the factors that affect an animal’s movement costs. However, in studies of animal locomotion energetics, the costs of movement may be measured, estimated and considered in manifold ways, which can lead to very different interpretations concerning the calculated energetics of the animal and/or the potential significance of its behaviour. In this opinion piece, we illustrate this issue by focusing on the interpretation of the energy expenditure of birds flying in so-called ‘cluster flocks’ in which many individuals move together in a loose association or flock, within which the birds may exhibit variable spacing and positioning over time. Several studies of homing pigeons *Columba livia* have suggested: that individuals will incur ‘considerable energy cost to flight in a tight cluster flock’ [7], that flying in cluster flocks is likely to come ‘at a cost for most individuals’ [8] and that ‘We know that flying in a flock comes at a cost in pigeons’ [9]. These conclusions are usually juxtaposed to those for the more structured line or V-formation forms of flight, in which theory predicts that individual birds should gain an active aerodynamic benefit and, therefore, an energetic advantage [10,11]. However, we will argue that the largely detrimental view of the energetic outcome for flying in cluster flocks is based on insufficient evidence and is potentially confounded by several factors:

(1) The commonly used kinematic proxy variables for mechanical power output, such as wingbeat frequency or dynamic body acceleration, do not reflect the dimensions and units for power [12–14], leading to unreliable determinations of changes in power.
(2) Body mass is an important influence on bird flight kinematics, but these effects may not scale isometrically. Therefore, experimental and comparative data where body mass differs between groups should be evaluated using both absolute and mass-specific terms.
(3) Flight speed and, therefore, total journey energy expenditure may be at least as important as instantaneous energy expenditure.

## 2. Estimating the instantaneous rate of energy expenditure

### (a) Little evidence of higher flight costs for pigeons flying in a cluster flock

The generally accepted view, originating from the study of Usherwood *et al.* [7] of homing pigeon flight, is that it is fundamentally energetically expensive for a bird to fly in a cluster flock compared to flying alone [7–9]. This is largely predicated on the analysis and interpretation of increases to wingbeat frequency (*f*_W_), up to around a mean 0.1 Hz of ‘residual’ change, compared to a mean value of *f*_W_ around 7.05 Hz (based on figs 2 and 3 of [7]) for pigeons freely flying above their loft, where *f*_W_ is treated as a kinematic proxy for mass-specific mechanical power—higher *f*_W_ is assumed to associate with greater instantaneous energy costs, all other factors taken into account [7]. However, using a single kinematic measure alone to estimate flight energetic consequences does not reflect the mass-specific units of instantaneous mechanical energy expenditure (m^2^ s^−3^, equivalent to W kg^−1^), which highlights its potential for oversimplification [12–14]. Various publications by Pennycuick [12,15,16] have emphasized and demonstrated the value of utilizing suitable variables and appropriate units within a framework of a dimensional analysis to resolve kinematic problems.

Despite Usherwood *et al.* [7] pointing out that the inertial energy costs of wing flapping are likely to be proportional to the product of *f*_W_ cubed and wingbeat amplitude (*Φ*_W_) squared, they do not utilize this relationship when estimating the energetic influence of flying in a flock based on body motion. This may be unfortunate, given that this proportionality is the same, in principle, as when calculating mass-specific mechanical body power in the vertical axis (*z*-axis) of motion (ms*P*_b,z_) as detected by a back-mounted accelerometer [13]. Body power is associated with the simple harmonic motion of a body, which is proportional to the product of *f*_W_ cubed and vertical body amplitude (*B*) squared. Thus, assuming body motion broadly approximates to a sinusoidal oscillation, then


(2.1) $$msP_{b,z}=4\pi^{2}B^{2}f_{w}^{3}.$$

**Figure 1.**
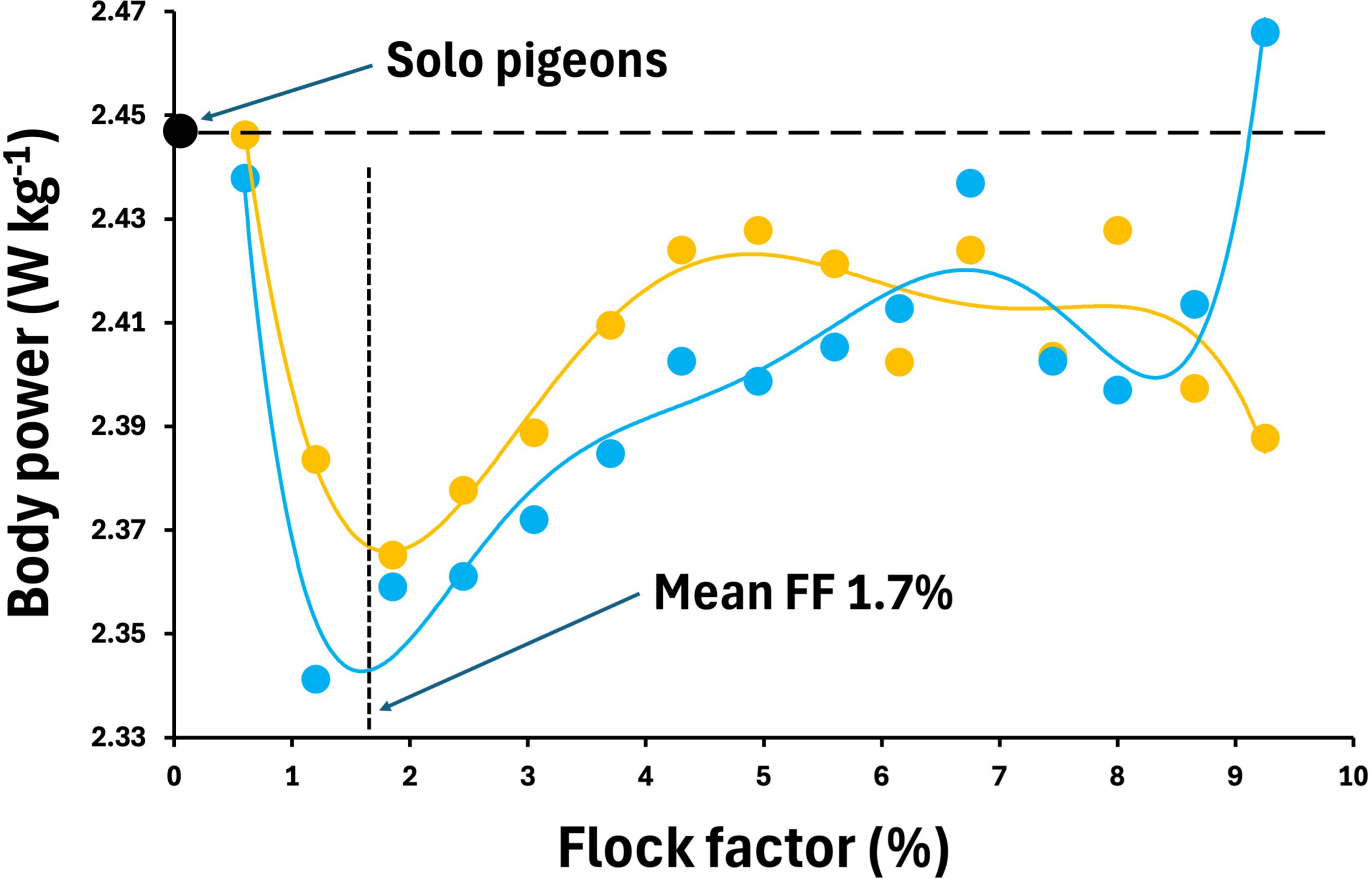
Estimates of mass-specific body power (ms*P*_b,z_) based on data taken from Usherwood *et al.* [7] (their figs 2 and 3), calculated using equation (2.1) in the text, and mean values *f*_W_ = 7.04 Hz and *B* = 13.3 mm estimated from [7]. Orange filled dots represent pigeons that are flying ahead of the flock and blue filled dots represent those flying behind other birds. The vertical dashed line shows the mean recorded flock factor (FF or flock density) recorded in the study at FF 1.7% [7].

Importantly, combining the two variables of *f*_W_^3^ and amplitude squared (whether *B*^2^ or *Φ*_W_^2^) results in an estimate of instantaneous mass-specific mechanical power output with the appropriate units. Clearly, there is the potential for *B* or *Φ*_W_ to negatively correlate with *f*_W_, so that an increase in *f*_W_ could be associated with a decrease in *Φ*_W_ or *B*, and therefore for the individual contribution of each of these kinematic measurements to the change in energetic outcomes to be at least partially compensated for or even cancelled out. In this regard, Usherwood *et al.* [7] did calculate values for change in *B* and showed that they decreased when flying in a flock (up to around a mean 0.45 mm of ‘residual’ change, compared to a mean value of *B* around 13.3 mm—based on their figures 2 & 3). If we add the mean residual changes in the values for *f*_W_ and *B* observed during flocking to the overall mean values (*f*_W_ = 7.05 Hz, *B* = 13.3 mm), we can calculate an estimate for the relative change in ms*P*_b,z_ by applying equation (2.1). As a result, we can reasonably conclude that pigeons flying in a typical density cluster flock generally fly with a *lower* value of ms*P*_b,z_ (mean ± s.d. flock ms*P*_b,z_ : 2.403 ± 0.03 W kg^−1^) than when flying alone (2.447 W kg^−1^), due to the relative reduction in *B*^2^ being greater than the relative increase in *f*_W_^3^ (figure 1). Furthermore, the pigeons flying behind other birds only ever exceeded the ms*P*_b,z_ of those flying alone when measured at the single highest ‘flock factor’ (FF) density recorded (FF ~9%, 2.466 W kg^−1^ [7]), an energetic increase of only ~0.78% and hardly ‘considerable’. Indeed, for most of the flock densities studied (mean recorded FF was only 1.7% [7]), the pigeons positioned at the back of the flock and, thus, potentially most affected by the movement and aerodynamic influence of conspecifics, are working no harder than those at the front and possibly a little bit less.

A study by Sankey & Portugal [17], entitled ‘When flocking is costly’, focused on pigeons flying in a cluster flock for nearly 100 min to assess potential changes in flight behaviour as the birds tired, which eventually resulted in slower flight speeds and a loss of flock coherence. The data from their figure 2 [17] reveal a decreasing trend in *f*_W_ (ranging from around 7.1 to 6.7 Hz) and an increasing trend in *B* (ranging from around 12.9 to 14.3 mm). Applying equation (2.1) indicates that the product of *f*_W_^3^ and *B*^2^ cancel out and, thus, there is no trend in estimated instantaneous ms*P*_b,z_ output over the course of the flight (estimated mean ± s.d.: 2.37 ± 0.16 W kg^−1^). Therefore, as in the study of Usherwood *et al.* [7], there is no convincing support for flock flight being inherently costly.

*B* is typically calculated by double integration of the vertical axis body acceleration or by dividing the root mean square of acceleration by $\sqrt{8}$π^2^*f*_W_^2^ [18]. This does inevitably add uncertainty around the resolution and accuracy of *B*. Sufficient data collection will increase the statistical power of the measurement and should produce a valid experimental variable. Additionally, the movement of the body during flapping flight results from many different aspects of wing motion, including angle of attack, adjustments to wing extension and wing area and the proportion of time in the upstroke compared to the downstroke. However, these factors will ultimately be integrated into the overall resultant movement of the body.

### (b) Empirical evidence shows that wingbeat frequency does not always positively correlate with the rate of energy expenditure

A recent study has shown that European starlings *Sturnus vulgaris* [19] flying as pairs in a wind tunnel can position themselves such that the bird following behind a leader has an energetic advantage. Flying downstream from the leader resulted in a modal *decrease* in net energy expenditure of up to 25% (measured using the ^13^C-labelled Na-bicarbonate method), and most importantly, this was despite an *increase* in *f*_W_. This result for starlings supports our re-analyses of Usherwood *et al.* [7] and Sankey & Portugal [17], by empirically demonstrating that interpreting energy expenditure based on changes in *f*_W_ alone is not likely to be sufficiently accurate or even qualitatively reliable. Interestingly, footage of wild starlings flying in murmuration flocks prior to roosting indicates that the structure of the ‘cluster’ flock is quite dynamic, with birds frequently placed side-by-side or primarily laterally spaced with respect to their nearest neighbour, such that their predicted aerodynamic influence would be predominately energetically neutral [20]. Furthermore, this does not rule out the possibility of choosing a more aerodynamically favourable spacing when performing commuting types of journeys [19]. Indeed, free-flight observations of flocks of dunlin *Calidris alpina* [21] indicate that positional spacings are highly dynamic between individuals and can briefly even resemble line-type formations which might be aerodynamically advantageous.

### (c) Effects of body mass on acceleration-based proxy estimates of mass specific and absolute instantaneous energy expenditure

Sankey & Portugal [9] reported a complex study addressing pigeon flock behaviour. In the supplementary material of their publication, they describe an experiment comparing pigeons of varied body mass and showed that heavier birds had a higher *f*_W_, which they interpreted as heavier birds expending energy at a higher mass-specific rate. However, as in the studies reported in §2(a) [7,17], those birds flying with a faster *f*_W_ again exhibited a compensatory lower value of *B*. Although Sankey & Portugal [9] point out that the effect size is larger for the change in *B* than for *f*_W_, they then focus solely on the increase in *f*_W_ as a proxy for mass-specific mechanical power, likely due to an erroneous belief that the mass-specific instantaneous cost of flight must be greater in heavier birds, especially as the naturally heavier birds also flew slightly faster. However, the mass-specific energetic consequences of additional mass may scale hypo-allometrically in flying birds [22]. Thus, for this type of experimental comparison we should utilize a proxy that can account for differences in body mass between individuals and, therefore, reflects both absolute and mass-specific instantaneous energy expenditure, such as ms*P*_b,z_ × body mass (Newton’s laws of motion show that absolute power = mass × acceleration × velocity, kg m^2^ s^−3^, equivalent to W). A simplified analysis of the relationships presented in their figure S1(D-F) indicates that a light pigeon (e.g. ~0.41 kg) would fly with a speed of ~16.5 m s^−1^, with a *f*_W_ of ~5.2 Hz and a *B* of ~20.5 mm, while a heavy pigeon (e.g. ~0.53 kg) would have a *f*_W_ of ~5.4 Hz, a *B* of ~19 mm and a speed of ~18 m s^−1^. Applying the ms*P*_b,z_ proxy of equation (2.1) and multiplying by body mass, we obtain an estimated instantaneous energy expenditure for absolute *P*_b,z_ of 2.333 W kg^−1^ × 0.41 kg = 0.957 W and 2.244 W kg^−1^ × 0.53 kg = 1.189 W for light and heavy birds, respectively. Thus, the more massive body of the heavier pigeon dampens its vertical axis body acceleration and *reduces* ms*P*_b,z_, even though absolute *P*_b,z_ has increased.

## 3. Total versus instantaneous energy costs of a flight—the importance of airspeed

### (a) Evidence from acceleration kinematics

While analyses of flock energetics have traditionally focused on instantaneous mass-specific costs, this may not always capture all the relevant aspects of energy expenditure, since the relationship between the instantaneous energy costs and the overall or accumulated energy cost of a given journey differs as and when flight airspeed changes (all other things being equal). The energy utilized over an entire flight is highly relevant, assuming a bird is aiming to minimize overall energy expenditure or optimize net energy gain [23,24].

**Figure 2.**
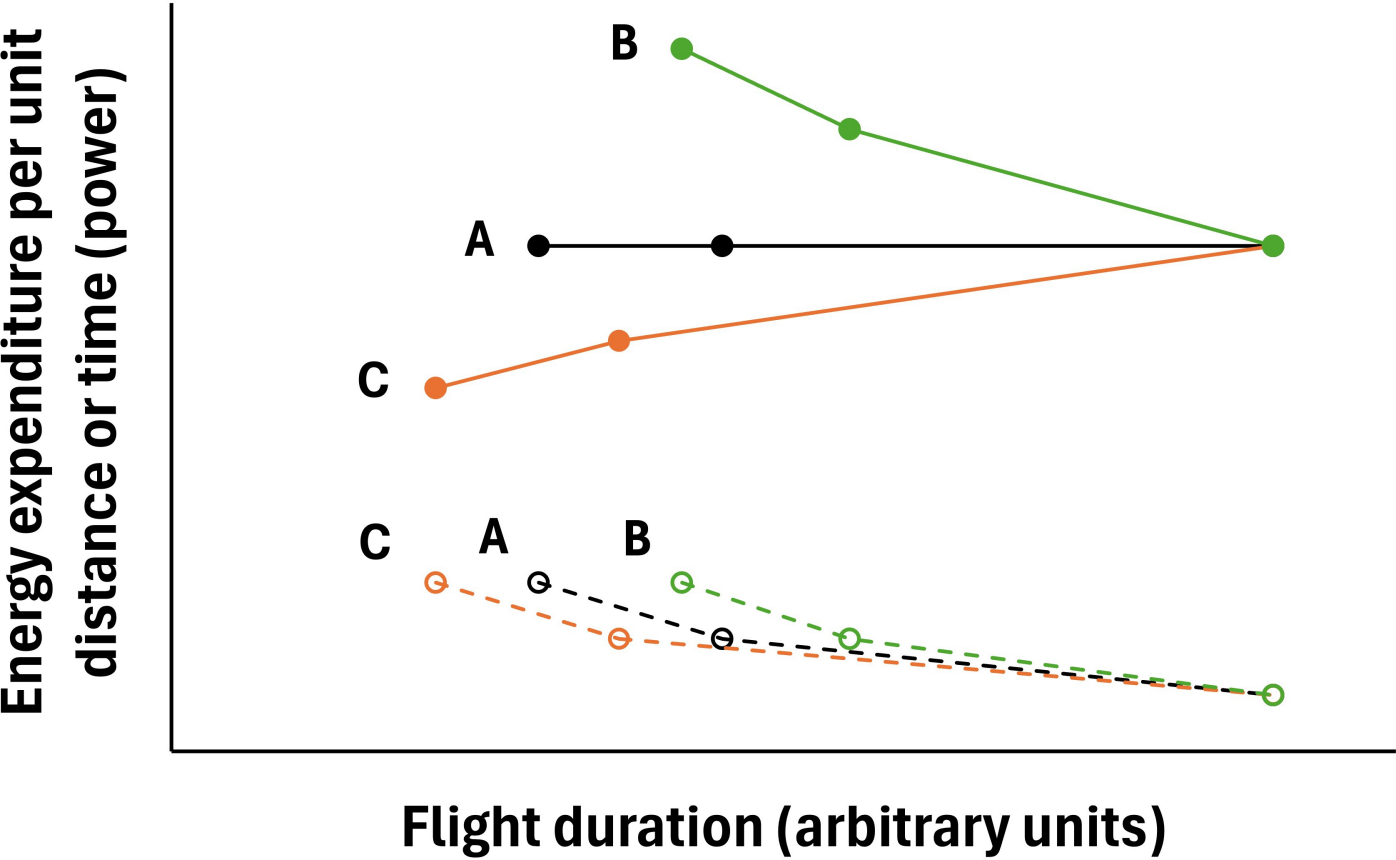
Theoretical relationships between energy expenditure during flight and time taken to fly a fixed distance. Open circles and stippled lines represent instantaneous rate of energy expenditure (power); filled circles and full lines represent total energy expenditure for the flight. (A) Black lines and symbols—if instantaneous rate of energy expenditure is linearly related to speed and thus inversely linearly related to the time to fly the fixed distance, then total energy expenditure per unit distance is invariant with flight duration; differences in instantaneous rate of energy expenditure do not result in differences in total accumulated energy expenditure. (B) Green lines and symbols—if instantaneous rate of energy expenditure is hypo-allometrically related to flight duration (i.e. flight speeds increase less than unitarily as instantaneous energy expenditure increases), then total accumulated energy expenditure increases with decreasing flight duration. (C) Orange lines and symbols—if instantaneous rate of energy expenditure is hyper-allometrically related to flight duration then, as flight duration decreases, total accumulated energy expenditure decreases.

In theory, increased instantaneous costs could result in total energy expenditure per unit distance travelled decreasing, increasing or remaining constant (figure 2). For example, and most pertinently, if a relatively small increase in instantaneous energy expenditure results in a relatively large increase in flight airspeed, such as when birds are flying at speeds somewhat above the nadir of their *U*-shaped power curve [25,26] (i.e. somewhat above those at which instantaneous energy expenditure is minimal), then total accumulated flight costs will be lower (figure 2C) assuming no change in route efficiency. Indeed, commuting pigeons typically fly at speeds above their minimum power speed (*V*_mp_), which can result in more economic journeys (i.e. lower total energy expenditure) [22]. Sankey & Portugal [9] provide a detailed discussion that hints to the fact that changes to the instantaneous energy costs of birds in the flock may be quite subtle, but that when different flocks fly at faster airspeeds they have a lower total number of wing beats and summed *B*. This would indicate that the total cost of the whole flight of the birds in these faster flocks is reduced and that they are, therefore, flying nearer their maximum range speed (*V*_mr_) [25].

**Figure 3.**
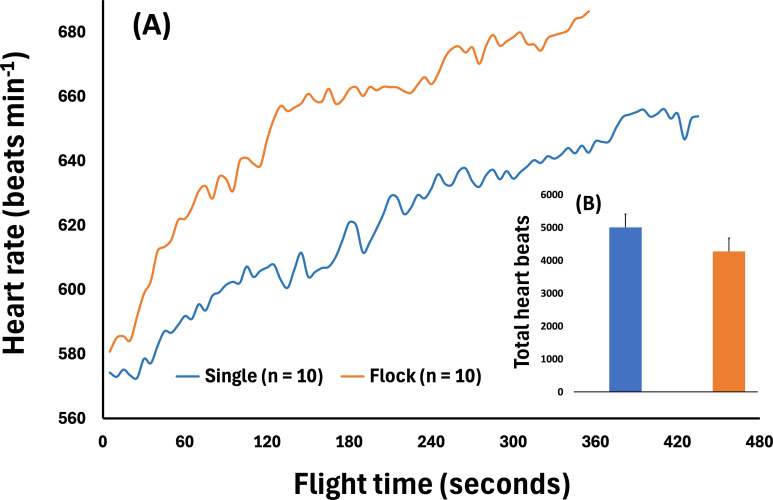
(A) Mean heart rate of 10 pigeons flown in a single flock (orange) and 10 pigeons flown singly (blue). Plot ends when less than 10 pigeons were in the air for each mode. (B) Histogram showing accumulated total heart beats for single and flock pigeons (mean±SD).

### (b) Evidence from heart rate measurement

We can empirically address some of our arguments regarding instantaneous and total flight costs by analysing data from our pilot experiment on homing pigeons flying 6 km to their loft, deployed with ECG data loggers [22] to determine their heart rates (figure 3A). Using heart rate as a proxy for the rate of energy consumption in pigeons is typically more robust than using *f*_W_, because cardiac stroke volume does not vary greatly once birds are exercising using their dominant mode of locomotion [27,28]. Moreover, there is a published calibration of heart rate for pigeons [22] and some other species of birds [28–31], which can provide an estimate for the rate of oxygen consumption ($\dot{V}$O_2_) or metabolic power. Of 32 instrumented birds, half were released as a flock and half were released singly. Twenty of the loggers recorded ECG suitable for analysis. The 10 pigeons in the flock (body mass 342−460 g) had a higher mean heart rate than the 10 single birds (body mass: 338−477 g; mean *f*_H_ ± s.d.: 652 ± 7.4 versus 621 ± 5.4 beats min^−1^, respectively, Student’s *t*-test, *p*‐value = 0.006; figure 3). This yields an estimated 10% lower rate for instantaneous energy expenditure for the single birds. However, the flock flew faster than the solo pigeons, having a shorter flight time back to the loft (355 ± 11.9 s versus 435 ± 9.2 s, Student’s *t*-test, *p*‐value < 0.001), at an estimated ground speed of 18.6 and 15.2 m s^−1^, respectively (assuming 90% efficient following of the straight-line distance). Thus, the higher *f*_H_ of the pigeons flying as a flock may be adequately explained by their higher flight speed.

The mean *f*_H_ for the flock birds only increased by a factor of 4.9%, while the mean flight time of the solo birds increased by 22.5%, with the result that the total heart beats accumulated over the flight journey was lower for the flocking birds (3855 ± 139 beats versus 4505 ± 117 beats). This represents a 14.4% decrease in total number of accumulated heart beats, which in turn, based on the conversion equation for scaled $\dot{V}$O_2_ = 0.048 *f*_H_^2^ ml min/*M*_b_^0.328^/*M*_h_^0.913^ (accounting for body mass and heart mass, respectively) [22,28], represents an estimated 10% decrease in total energy expenditure. Thus, the overall flight of the pigeons in the flock was more economic per unit distance, despite having a higher instantaneous *f*_H_, presumably as the faster flying birds brought them closer to their *V*_mr_. Pennycuick [25] calculated *V*_mp_ of pigeons at 8−9 m s^−1^ and *V*_mr_ at around 16 m s^−1^. More recent studies suggest the use of lower default values for the parasite drag coefficient [32,33], yielding a *V*_mp_ of around 13 m s^−1^ and, thus, a *V*_mr_ > 16 m s^−1^. These higher values for *V*_mp_ and *V*_mr_ are consistent with our measurements of pigeon flight airspeeds and total accumulated heart rate. The estimated 12% increase in the accumulated total flight costs for pigeons flying alone (figure 3B) is higher than the 7% increase recorded from pigeons when carrying 10% extra body mass [22]. Thus, it seems parsimonious to conclude that, after accounting for the variation in speed, *f*_H_ would not differ significantly between the flock and lone-flying pigeons.

Various studies have reported that pigeon flocks sometimes fly faster and/or with shorter journey times than birds flying solo [34]. While the latter finding may arise due to the advantage of shared navigational decision making leading to a higher route efficiency, any variation in the distance flown was controlled for in the study by Sankey & Portugal [9]. Thus, their results are fully consistent with our heart rate data (i.e. they recorded a drop in total *f*_W_ and summed *B* for the whole flight, which parallels the drop in total heart beats recorded in our pilot study). Furthermore, the confounding hypothesis of an increase in route efficiency does not explain the higher heart rates exhibited by birds in the flock because if they were flying at the same speed but along a straighter route we would reasonably expect the same metabolic rate per unit time.

## 4. Conclusions

Instantaneous rates of energy expenditure and total journey costs, though related, represent quite different selective pressures on an animal, and they may trade off against each other, leaving the researcher with much to ponder when evaluating flight costs during longer journeys. Clearly, measuring the energy costs of free-flying animals is challenging, and the various proxies that have so far been utilized each have their strengths and weakness [28,35–37]. Our re-analysis of the published results for estimates of the mechanical energy expenditure of pigeons during flight [7,9,17] is supported by our heart rate measurements. These results suggest that for birds flying in a typical ‘cluster’ flock energetic costs are not greater than when flying solo. Thus, we suggest that birds may use sufficient behavioural and positional plasticity within cluster flocks that spacing arrangements and their associated flight kinematic gaits do not, on average, incur extra costs compared to flying alone and may even result in a net energetic advantage. Indeed, using data reported by Usherwood *et al.* [7] and graphed in figure 1, moderate flock densities for free-flying pigeons of between 1% and 6% appear to have a mechanical energetic advantage over solo flying, while the lowest overall flight costs coincide with the mean recorded flock density of 1.7% and point towards an optimal flock arrangement.

## Data Availability

Data are available via electronic supplementary material (BL Figure 1 data supplementary; BL Sankey and Portugal data; BL Figure 3 data supplementary). Supplementary material is available online [38].
